# 

**Published:** 1904-01-02

**Authors:** 


					THE HOSPITAL. ^5 MR r ? j\Staddjan$' pf highest purity."?Lancet. January 2nd, 1904.
CADBURY'I jjfift U CADBURY'S
nnnn a UMIN. J J Ml; 4- JMCiMl
COCOA UHM- A JiriW? M p> JEL> COCOA
"A PERFECT fOOD." NO DRUGS OR ALKALIES USED. ""
No. 901. Vol. XXXV. [aNgSfpearSl SATURDAY, JAN. 2ND, 1904. [S^^ript^n Term,,] PR|CE THREEPENCE.

				

